# Impacts of Parasites in Early Life: Contrasting Effects on Juvenile Growth for Different Family Members

**DOI:** 10.1371/journal.pone.0032236

**Published:** 2012-02-24

**Authors:** Thomas E. Reed, Francis Daunt, Adam J. Kiploks, Sarah J. Burthe, Hanna M. V. Granroth-Wilding, Emi A. Takahashi, Mark Newell, Sarah Wanless, Emma J. A. Cunningham

**Affiliations:** 1 Institute of Evolutionary Biology, University of Edinburgh, Edinburgh, United Kingdom; 2 Centre for Ecology and Hydrology, Penicuik, Midlothian, United Kingdom; 3 Netherlands Institute for Ecology, Wageningen, The Netherlands; University of South Alabama, United States of America

## Abstract

Parasitism experienced early in ontogeny can have a major impact on host growth, development and future fitness, but whether siblings are affected equally by parasitism is poorly understood. In birds, hatching asynchrony induced by hormonal or behavioural mechanisms largely under parental control might predispose young to respond to infection in different ways. Here we show that parasites can have different consequences for offspring depending on their position in the family hierarchy. We experimentally treated European Shag (*Phalacrocorax aristoteli*) nestlings with the broad-spectrum anti-parasite drug ivermectin and compared their growth rates with nestlings from control broods. Average growth rates measured over the period of linear growth (10 days to 30 days of age) and survival did not differ for nestlings from treated and control broods. However, when considering individuals within broods, parasite treatment reversed the patterns of growth for individual family members: last-hatched nestlings grew significantly slower than their siblings in control nests but grew faster in treated nests. This was at the expense of their earlier-hatched brood-mates, who showed an overall growth rate reduction relative to last-hatched nestlings in treated nests. These results highlight the importance of exploring individual variation in the costs of infection and suggest that parasites could be a key factor modulating within-family dynamics, sibling competition and developmental trajectories from an early age.

## Introduction

Environmental and social conditions experienced at critical early life stages can impact juvenile growth and development in vertebrates, with potentially long-lasting effects on health and performance [1], [2], [3], [4]. In birds, stressful rearing conditions such as food and nutrient limitation, inclement weather and sibling competition can depress nestling growth rates [5], [6], [7], [8], and reduced size and/or body condition at fledging has been shown across species to negatively impact post-fledging survival and recruitment success [9], [10], [11], [12]. Experimental brood enlargements in zebra finches (*Taeniopygia guttata*) also show that developmental stresses on nestlings can carry through to affect patterns of reproductive investment in adulthood [13], phenotypic characteristics of offspring produced [14], and the reproductive success of these offspring [15].

Parasitism is a key factor affecting individual performance, population dynamics and life-history evolution in birds [16], [17], [18]. Young birds are potentially more severely affected by parasitic infection than adults as a result of a less efficient immune system, which is not fully developed at hatching [19], [20], and exposure to nest-dwelling parasites [21]. While maternal transfer of immunity provides some degree of protection, primary immune responses launched by nestlings upon initial contact with a parasite can be weak and take longer to activate than responses following subsequent contacts [22]. Exposure to parasitism early in ontogeny can have delayed fitness consequences, both in terms of future survival, but also potential mating success (e.g., impacts on male song duration) [23] and fecundity (e.g., clutch size and lifetime reproductive success) [24]. More immediately, however, parasitism can alter early developmental trajectories [25], [26], [27], [28], as resources otherwise allocated to growth are diverted to fight infection [29].

While it is clear that parasitism can have both immediate and delayed impacts on avian hosts [19], it is less well-understood whether nestlings differ in their susceptibility. Hatching asynchrony within broods, generated via hormonal or behavioural mechanisms largely under parental control, often leads to nestling size hierarchies being established in birds. Late-hatching nestlings are typically smaller and competitively inferior to their older, larger brood-mates [30], predisposing them to fledge in poorer condition [31], [32]. Asynchronous hatching might be adaptive (from a parental fitness perspective) if it ensures a core brood has optimal survival chances should food shortages limit the ability of parents to provide for all young [33], [34]. Even in years where all young can be raised, however, marginal young might still be more vulnerable to parasites. For example, Saino et al. [31] found that barn swallow (*Hirundo rustica*) nestlings hatching late within a brood had higher immunoglobulin concentrations and higher intensity of T-cell mediated immunity, compared to early-hatched nestlings. They hypothesized that this reflected greater investment in immunity by late-hatched nestlings, as a result of potentially greater exposure or susceptibility to parasitic infection (although differential maternal allocation might also explain some of the differences) [31].

Within-family variation in responses to parasitism could also be influenced by nestling sex (or attributes that differ between the sexes such as size) but the direction of these effects are difficult to predict *a priori*. On the one hand, males might be more susceptible to parasites as a result of reduced immunocompetence linked to elevated testosterone [35]. For example, Tschirren, et al. [36] found that body size and mass of male, but not female, great tit (*Parus major*) nestlings approaching fledging was significantly lower in nests where ectoparasite loads had been experimentally increased compared to uninfested nests. Males also exhibited significantly reduced cell-mediated immune responses compared to females, suggesting lower immunocompetence [36], see also [37]. Alternatively, the impacts of parasites might be less severe for male nestlings if their ability to compete for food under stressful conditions (e.g., high parasite burdens) is less strongly affected than that of female siblings, particularly in sexually-dimorphic species where males are bigger [38].

We experimentally tested whether offspring within broods differ in their responses to parasitism by administering a broad-spectrum anti-parasite drug (which affects both ectoparasites and endoparasites) to all brood members and monitoring individual growth and survival during the developmental period in the nest. The experiment was replicated across two breeding seasons. The European shag (*Phalacrocorax aristotelis*) provides a good model system in which to explore these effects. First, shags frequently suffer from heavy infections of nematode gut parasites as well as infestations of ectoparasitic feather lice [39], [40], [41]. We have previously demonstrated that parasites play a major role in both driving seasonal declines in breeding success in our study population and in limiting the ability of females to rear costly male offspring [41] (males are ∼20% heavier at fledging than females). Second, shag nestlings have a variable start in life depending on their hatching position. In clutches of 3 (the modal clutch size in our study population), first and second nestlings hatch within 24 hours of each other and the third nestling usually hatches 2–3 days after the second-hatched. This sets up a pronounced initial size hierarchy among siblings [42]. We examined whether parasites negatively affected the growth and survival of shag nestlings, and whether certain brood members were disproportionately affected in relation to their position in the brood hierarchy and sex.

## Materials and Methods

### Ethics statement

The work was conducted under UK Home Office licence and was in accordance with their guidelines for animal welfare. All necessary steps were taken to minimise animal suffering in this study.

### Study population

The study was conducted during the breeding seasons of 2006 and 2007 at a breeding colony of approximately 500 shags on the Isle of May, south-east Scotland (56°11′N, 02°33′W). Shags can lay up to 4 (very rarely 5) eggs but have a modal clutch size of 3 (∼85% and 70% of nests monitored in 2006 and 2007, respectively, had a clutch size of 3). We therefore focused on 3 egg nests for this experiment. Parents initiate incubation before the clutch is complete - either immediately after the first egg is laid, or shortly after the laying of the second egg. This behaviour induces a hatching asynchrony within clutches: the first and second-laid eggs hatch usually within a day of each other (60% of sampled nests hatched first and second eggs on the same day, the rest hatched them within 48 hours of each other), while the third-laid egg hatches anywhere from 1 to 4 days later (75% hatched 2–3 days after second eggs). Nestlings are fed immediately by parents, so early-hatching nestlings get a growth head-start over later-hatched siblings. Consequently, distinct size hierarchies usually develop within broods during the early phase of nestling rearing. When the oldest nestling is ∼10 days old, the most obvious size disparity is between the smallest, last-hatched nestling and its two older siblings [42].

### Defining size hierarchies

**Figure 1:**
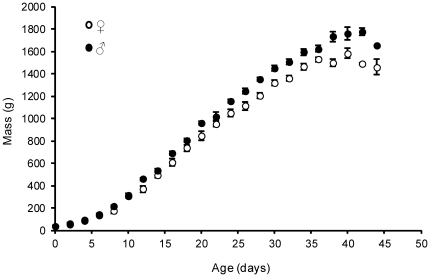
Growth curves for male (solid circles) and female (open circle) nestlings in 2006. Data points are mean (±SE) mass measures for each nestling age, binned into two day periods here for illustrative purposes. Growth is approximately linear from day 8 to 30. Note that female data points from age 0 to 6 are obscured by male data points at those ages (i.e. no difference).

We defined size hierarchies within broods when oldest nestlings were approximately 8–12 days old (hatch dates and therefore nestling ages were known to within ±2 days). This age marks the beginning of the linear phase of growth [43] (see Fig. 1). The heaviest nestling in the brood at this stage was labelled the A nestling (mean initial mass = 489±25 g [SE], n = 42), the second-heaviest the B nestling (mean initial mass = 428±22 g, n = 42), and the lightest the C nestling (mean initial mass = 287±23 g, n = 42). The ranking of A, B and C nestlings according to initial mass differences typically (but not always) persists and in some cases the absolute differences become magnified over the ∼50-day period when nestlings are being fed by parents in the nest [44]. Shags are also sexually dimorphic; male nestlings grow faster and reach higher peak masses [43] by fledging than females (Fig. 1).

### Parasites affecting shags

European shags are parasitized by gastro-intestinal nematodes [39], principally in our population anisakids from the genus *Contracaecum* [41]. Although usually sub-lethal, these parasites compete with the host for nutrients and trigger costly immune responses [45]. Nestlings acquire worms when being fed by parents, either by receiving infected fish, or via direct transmission of larval-stage and adult nematodes that become dislodged from the gut of the parent during the regurgitation process. *Post-mortem* examination of nestlings in 2005 and 2006 revealed that nestlings often harbour tens to hundreds of these nematodes in their alimentary canals, in particular the proventriculum (T. Reed, unpublished data). Nestling shags also suffer from ectoparasitic louse (*Eidemanniella pellucida*) infestation. While a previous study found no discernible effect of these parasites alone on nestling growth and survival [40], they might impact host performance in combination with endoparasites. A large sample of broods was treated with a broad-spectrum anti-parasite drug, ivermectin [46], which either removes gut parasites and ectoparasites within the 24-hour period following treatment, or reduces their numbers or activity [47], [48]. Data on the efficacy of the treatment were not available for the years of the study, but faecal egg counts conducted during the 2010 breeding season (which can detect the eggs of parasites such as *Contracaecum* that mature and reproduce in the host) showed significantly lower parasite prevalence in nestlings that were treated with a similar dose of ivermectin at the same age as those in this experiment compared with control nestlings 2–3 weeks post-treatment (generalized linear mixed model, with nest as a random effect: treatment effect: *z* = −2.970, *P* = 0.003, n = 167 nestlings sampled in 43 nests, no significant effect of nestling age at dosing; H. Granroth-Wilding unpublished data). The number of parasite eggs detected post-treatment was also lower for ivermectin-treated nestlings compared to controls, controlling for initial burdens (z = −3.150, *P* = 0.002, n = 98 nestlings sampled in 42 nests). By comparing growth of treated nestlings to that of untreated controls, we were able to examine the overall impact of natural parasite levels in this population on nestling growth rates.

### Experimental design

Experimental nests - each containing broods of three nestlings - were assigned randomly to either a treated group (n = 20 nests in 2006, n = 17 in 2007) or a control group (n = 18 in 2006, n = 17 in 2007). Within nests, all 3 brood members received the same treatment. Nestlings in the treated group were administered approximately 0.05 ml of 1% aqueous solution ivermectin (Panomec®, Merial Ltd., UK) subcutaneously when the oldest nestling in the brood was approximately 8–12 days old. In 2006, the control group was a random sample of undisturbed nests, in which nestlings did not receive the ivermectin treatment, and therefore were presumed to suffer from natural levels of parasitism. In 2007, nestlings in control nests were sham-treated with 0.05 ml of distilled water, to control for possible negative effects of subcutaneous injection. Treated and control nests in both years were matched for hatching date (the distributions of hatching dates of first-hatched eggs were not significantly different between each group: two-sided Kolmogorov-Smirnov test pooling data from both years, n = 61 nests where hatch dates known: *P* = 0.972) and location in the colony where possible. The magnitude of the initial mass difference between A and B nestlings was not significantly different between treated and control groups (mean difference for treated broods = 67.62±10.52 g (SE), mean difference for control broods = 58.50±10.98 g; *F_1,39_* = 0.351, *P* = 0.557), nor was that between C nestlings and the average of A and B (mean difference for treated broods = 179.71±13.76 g, mean difference for control broods = 162.25±16.15 g; F_1,39_ = 0.668, P = 0.420). Faecal eggs counts conducted in 2010 found no significant difference in parasite prevalence between A, B and C nestlings prior to treatment (GLMM with binomial errors and nest as a random effect: effect of nestling rank: *z* = −0.531, *P* = 0.595; n = 62 nestlings sampled in 34 nests; nestling age, sex and hatch date effects not significant; H. Granroth-Wilding unpublished data), nor in the number of parasite eggs detected (GLMM with Poisson errors and nest as a random effect: no difference between A and B chicks: *z* = −0.411, *P* = 0.681, or between B and C chicks: *z* = −0.729, *P* = 0.4670; n = 62 nestlings sampled in 34 nests; H. Granroth-Wilding unpublished data). Sex differences in nestling growth rates are apparent in this species from an early age [43], [49]. Nestling sex was determined using molecular techniques from blood taken soon after hatching under UK Home-Office license (see Ethics Statement). Brood sex ratios at the beginning of the experiment were not significantly different between treated and control nests (control nests = 45.8% males, treated nests = 49.1% males; binomial-test of proportions: *P* = 0.736, n = 66 nests where sex of all brood members known) or between years (2006 = 50.0% males, 2007 = 46.0% males, binomial-test of proportions: *P* = 0.558, n = 66 nests).

In 2006, nestlings were weighed (to the nearest 0.1 g up to 200 g; to the nearest 2.5 g from 200 to 1000 g; to the nearest 10 g over 1000 g) approximately every 4 days from hatching to close to fledging (mean age of final weighing = 35.7±0.6 days). Nestling growth rate was estimated as the gradient (slope) of mass change during the linear phase of growth (nestling age 8–30 days; Fig. 1). In 2006, all nests but one (where the C nestling died soon after the first measurement was taken) produced 3 nestlings that survived long enough to be weighed on at least two separate occasions (the minimum to obtain an estimate of growth rate). In 2007, nestlings were weighed only twice: first when nestlings were ∼8–12 days old, and again towards the end of the linear phase of growth (mean age of final weighing in 2007 = 29.3±0.2 days). Nestling mortality was high in 2007: 11 nests failed to produce a single nestling that survived long enough to be weighed twice, leaving 23 (of the original 34) where growth rates could be estimated for at least one nestling in the brood (13 controls and 10 treated). Of these, only 5 nests produced 3 surviving nestlings. We used the full dataset (n = 37 treated nests, n = 35 control nests) to analyse nestling survival to fledging (i.e., fledging success; see below), but when analysing growth rates, we initially restricted our analysis to nests where all 3 nestlings survived to age 30 days. This yielded a final sample size of 20 control nests (17 in 2006 and 3 in 2007) and 22 treated nests (20 in 2006 and 2 in 2007). We then repeated the growth rate analysis using the larger dataset i.e., all nests where at least one nestling survived long enough to be weighed twice (31 controls and 30 treated) and included brood size as a covariate to account for variable family sizes and ensure our results were not biased by data restriction.

Growth rate estimates are potentially sensitive to the number of data points per nestling during the linear growth phase. To test this, the 2006 mass data were restricted to 2 measures per individual – an initial and a final weighing – and growth rates were recalculated this way (emulating the fact that growth rate estimates in 2007 were based on two widely spaced mass measurements). The correlation between growth rates estimated this way and growth rates estimated using the full mass data was very high (*r* = 0.944, *P*<0.001). We also repeated analyses using 2006 growth rates calculated with only 2 data points, and model results were qualitatively and quantitatively similar to analyses based on growth rates calculated with the full data (results not shown).

### Statistical analysis

Linear mixed-effects models (LMMs) with restricted maximum likelihood estimation (fitted using the R package lme4) were used to examine the effects of treatment, position in the size hierarchy (factor with 3 levels: A, B or C), sex and their interactions on nestling growth rates, accounting for non-independence of nestlings from the same nest by including nest as a random effect. Year was also modelled as a random effect.

Seasonal effects and associated differences in parental capabilities, which might affect nestling growth rates, were controlled for by including hatch date of first nestlings as a covariate in the analysis (early breeding parents tend to raise more chicks, associated with their often superior foraging capabilities and/or better environmental conditions early in the season) [41], [50]. Brood sex composition can also affect offspring growth [51]. The number of brothers (for each focal nestling in a brood) was therefore included as a three level factor (0, 1 or 2 brothers) in the analysis to control for potential uneven sex composition across broods. We started with a full model including all two and three-way interactions between treatment, position in the size hierarchy, and sex, as well as main effects of laying date , sex composition, and mass-at-treatment (see below). We then used a backwards stepwise model simplification procedure, sequentially removing non-significant terms (*P*>0.05) using Type III tests starting with higher-order terms, to yield the minimum adequate model. *F* and *t* tests are an approximation in LMMs because the denominator degrees of freedom are not well defined [52]. *P*-values for these tests were instead generated through an iterative Markov-Chain-Monte-Carlo sampling procedure, with 1×10^4^ iterations, implemented in the R package language [53].

All brood members were treated on the same day, rather than at a fixed age for each nestling, to maintain the natural brood size hierarchy under normal conditions and to minimise disturbance at the nest. With this experimental design, C nestlings were treated at a lower mass than their A and B siblings, as they were two to four days younger and correspondingly lighter. Mass-at-treatment was included as a covariate in the main analyses, however, to control statistically for variation in initial mass among nestlings, which might have affected their subsequent growth rates.

Post-hoc analysis to test whether (a) growth rate differences between C chicks and the average of A and B chicks and (b) within-brood coefficients of variation (CV) in growth rates were significantly different between treatments were also performed, using unequal variance *t*-tests [54]. We also fitted generalized linear mixed-effect models (GLMMs) to test for differences in nestling fledging success (a binary variable), including the same fixed and random effects as for the growth rate models and using the glmer function in the R lme4 library. All statistical analyses were performed in R version 2.10.

## Results

### Nestling growth rates

**Figure 2:**
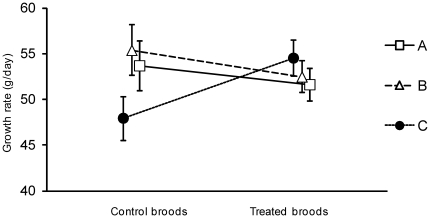
Mean growth (±SE) of nestlings in control and treated broods, showing differences between A, B and C nestlings. The data points have been slightly offset to allow the standard error bars to be clearly distinguishable.

Overall, there was no significant effect of the ivermectin treatment on nestling growth rates (Table 1; main analysis based on nests where all 3 three nestlings survived). However, growth rates of different brood members responded to the treatment in different ways, as evidenced by the significant interaction term between treatment and position in the size hierarchy (Table 1, Fig. 2). In control broods, A and B nestlings grew at the same rate but C nestlings grew slower (Fig. 2). In treated broods, however, C nestlings achieved similar or slightly better growth than their older siblings. A and B nestlings grew slower in treated broods compared to control broods (Table 1, Fig. 2). C nestlings were no more likely to be male in this study (45.4% male, n = 66 nests where sex known; binomial test for unequal sex ratio: *P* = 0.539). Within-brood differences between the growth rates of C nestlings versus the average of A and B nestlings were lower for treated nests (mean difference between A/B and C for treated nests = 2.44 gday^−1^; control nests: −6.60 gday^−1^; unequal variance *t*-test: *t = *2.42, *df* = 32.15, *P* = 0.021). The overall CV in growth rates (i.e., the within-brood standard deviation divided by the mean) was slightly lower for treated nests, but this difference was not significant (average CV for treated nests = 0.13; control nests: 0.16; *t = *−0.77, *df* = 28.15, *P* = 0.45).

**Table 1:** Summary of the minimum adequate linear mixed-effects model of nestling growth rates, fit using the lmer function in the R package lme4.

Sample sizes: n = 122 observations (excluding 4 nestlings where sex unknown).					
Groups: nests = 42, years = 2 (different sets of nests in both years).					
Random effects	Variance component				
Nest	13.417				
Year	16.916				
Residual	67.768				

Fixed effects	Estimate	SE	*df*	*t*	*P*
**Intercept (control female A nestlings)**	48.505	3.877		12.511	
**Treatment (dosed)**	−1.873	3.758	1	12.408	0.511
**Sex (male)**	5.217	1.619	1	3.223	**0.002**
**Position in size hierarchy (B)**	2.261	2.703	2	0.837	0.405
**Position in size hierarchy (C)**	−4.552	2.767		−1.645	0.103
**Treatment×position in size hierarchy (B)**	−1.007	3.684	2	−0.273	0.785
**Treatment×position in size hierarchy (C)**	7.659	3.705		2.067	**0.041**

P values were obtained using an MCMC routine in the R package languageR.

When the same growth analysis was re-run using data from nests in both years that produced at least one nestling, the results were qualitatively and quantitatively similar. Running the same minimum adequate model identified using the restricted dataset (see Table 1), but including brood size as a covariate, the treatment×position in the size hierarchy interaction remained significant (*F* = 3.493, *P* = 0.015). This was also true if the data were restricted to 2006 only and nests where all 3 nestlings survived (treatment×position in the size hierarchy interaction: *F* = 3.805, *P* = 0.033). Thus, the overall conclusion that treated C nestlings grew faster than control C nestlings appeared robust to data restriction.

Male nestlings grew significantly faster than females (Table 1), but there was no significant interaction between sex and treatment (*F* = 0.102, *P* = 0.750). Male C nestlings appeared to grow just as fast as male A and B nestlings (mean growth of male A nestlings 54.08±2.33 gday^−1^; male B nestlings 55.71±1.64 gday^−1^; male C nestlings 56.45±1.54 gday^−1^), although the sex×position in size hierarchy interaction was not significant (*F* = 0.87, *P* = 0.432; mean growth of female A nestlings 50.54±1.72 gday^−1^; female B nestlings 51.96±1.77 gday^−1^; female C nestlings 47.76±2.34 gday^−1^), nor was the three-way interaction with treatment (treatment×sex×position in size hierarchy: *F* = 1.19, *P* = 0.295). There was no effect of brood sex composition (*F* = 0.147, *P* = 0.868), or hatch date (*F* = 0.035, *P* = 0.955) on nestling growth rates.

Mass-at-treatment did not have a significant effect on nestling growth rates (*F = *0.430, *P = *0.649). To further explore whether the observed growth rate differences among treated nestlings with respect to position in the size hierarchy was confounded by mass-at-treatment differences, we compared the minimum adequate model identified in Table 1 with the same model but where ‘mass-at-treatment’ was substituted for the term ‘position in the size hierarchy’. The results showed that the model containing a main effect of position in the size hierarchy and its interaction with treatment had much stronger relative statistical support, as assessed by the Akaike information criterion (AIC), than the model with mass-at-treatment and its interaction with treatment (ΔAIC = −12.06).

### Nestling fledging success

Overall, there was no significant effect of the ivermectin treatment on nestling fledging success (controls: 76.0%, dosed 74.5%; *t* = 1.17, *z* = 0.103, *P* = 0.918). Male nestlings had marginally significantly higher fledging success than females (males: 97.4%, females: 85.4%, *z = *2.55, *P = *0.096). Highest fledging success was recorded amongst A nestlings (83.3%), followed by B nestlings (76.1%) and then C nestlings (63.6%) (A versus B: *z = *−1.671, *P = *0.095; A versus C: *z = *−2.085, *P = *0.037). None of the two-way interactions involving treatment, sex and position in the size hierarchy were significant.

## Discussion

While we found no overall differences in growth rates or fledging success between treated and untreated broods, treatment with a broad spectrum anti-parasite drug reversed the patterns of growth within a brood. Last-hatched treated nestlings grew significantly faster, often at the expense of the growth rates of their initially larger siblings, suggesting brood members are affected by parasitism in different ways. These growth rate differences were not being driven by a potential sex bias in hatching order [55], given that last-hatched nestlings were no more likely to be male (which might have resulted in them having different growth rates or susceptibility to parasitism). These findings demonstrate the importance of considering individual variation in responses when assessing the full impact of parasitism on host fitness [56].

There are a number of reasons why parasitism might generally be expected to have different effects on brood members that are unlikely to be mutually exclusive. Studies on other birds have found that variable impacts of infection on offspring might arise indirectly via post-laying parental effects. Knowles *et al* [57], for example, found that medicating parents against avian malaria parasites increased breeding success in blue tits (*Cyanistes caeruleus*) and that this effect appeared to be largely driven by reduced within-brood inequality in hatching success and fledging mass, allowing marginal offspring to be raised more successfully. In birds, last-hatched offspring generally show greater variance in growth and survival and are considered to be more susceptible to adverse environmental conditions than earlier-hatched, or ‘core brood’, nest mates [57], [58]. The production of marginal offspring (usually by means of hatching asynchrony), which can be provisioned if food is plentiful but are often neglected when food is scarce, is thought to represent an adaptive parental strategy to unpredictable resource fluctuations [34], [58], [59]. The effects of parasitism on parental ability alone might play a key role in determining the fate of different brood members. However, while we previously found that treating adult shags with ivermectin affected male and female offspring to different extents, we found no effect of anti-parasite treatment in adults on the growth rates or survival of nestlings associated with their brood order [41]. In the present study, offspring were treated against parasites rather than parents, but similar to the findings of Knowles *et al.* [57], in which parents were treated, anti-parasite treatment in chicks resulted in reduced within-brood inequality in offspring growth (see Fig. 2). This suggests that offspring responses to infection might directly mediate some of the negative effects of parasites on host breeding success. In our case, this was driven by reduced differences in the growth rates of marginal (i.e., C) nestlings compared to those of core (i.e., A and B) nestlings, rather than an overall reduction in within-brood growth variation in treated nests. The treatment did not appear to differentially affect the survival of marginal and core nestlings; however, small differences in fledgling mass could translate to large differences in recruitment probability [9], [10], [11], [12]. Core brood nestlings in treated nests also grew slower than marginal nestlings, suggesting anti-parasite treatment either had direct negative effects on core nestlings, or the improved growth of marginal young came at the expense of reduced growth of core young. The latter could occur if parental provisioning behaviour was invariant with respect to parasite treatment (e.g., if the total amount of food delivered to nestlings was similar for treated and control nests). Alternatively, parents might adjust provisioning rates in relation to offspring needs (e.g., as signalled by begging intensity) [60], in which case anti-parasite treatment might have improved the ability of marginal young to compete with core young when soliciting feeds from parents. We do not have data on parental provisioning behaviour in this study and so could not distinguish the relative roles of flexible parental provisioning versus direct effects of parasites on offspring physiology as potential drivers of the observed growth rates differences, although clearly this deserves future attention.

Another possibility is that mothers might actively or passively ‘assign’ resources differentially among family members at the pre-laying stage, which could subsequently affect nestling growth patterns in response to infection. This could include a lower investment in protective maternal immunity to the non-core brood and/or variation in hormonal (e.g., androgen) levels among nestlings as a mechanism for generating the brood hierarchy, which could hinder responses to parasitism as a side-effect [61], [62], [63]. These differences in parental allocation might cause brood members to vary in their ability to cope with direct costs associated with a heavy parasite burden; in particular, marginal offspring might be predisposed to pay a higher physiological cost for a given infection level or be more susceptible to infection and therefore carry more parasites. Faecal egg count data from the 2010 breeding season suggests that endoparasites are equally prevalent in 10–12 day old core and marginal offspring (H. Granroth-Wilding unpublished data), although there might still be differences in ectoparasite burdens. Work is on-going with this study population to examine variation in nestling infection levels and immunocompetence in relation to brood order, sex and other factors. Somewhat counter intuitively, studies on passerines have revealed that last-hatched nestlings often have higher immunocompetence than core brood members, despite being in poorer condition [31], [64]. These studies could not conclusively determine whether immunity differences between junior and senior nestlings were influenced by parental allocation patterns. However, in an experimental manipulation with blue tits in which first-laid eggs were forced to hatch last, Mainwaring, Dickens & Hartley [65] found no evidence for maternal effects on offspring growth rates, which might have occurred through any of the above mechanisms. Our study suggests that exposure to parasites post-hatching constitutes an important, but often overlooked, environmental factor contributing to within-brood variation in growth patterns.

Parasite host choice has also been hypothesised to influence differences in the response of different brood members to parasitism, for example if parasites selectively choose young with lower defences (the ‘tasty chick hypothesis’) [66]. This mechanism is most relevant in systems where ectoparasites have the greatest fitness effect on hosts and parasites can move easily between nestlings. Shags in our study population are commonly infested with ectoparasitic feather lice [40]; however, host-selection behaviour by endoparasites cannot be driving the effects observed in this study, as gut parasites are simply transferred in food meals by parents and are not free to move between hosts within the nest. While we cannot exclude the possibility that within-brood growth variation in shags is affected by ectoparasite preferences, a previous study found no impact of ectoparasites on nestling growth rates or survival in this population [40]. Recent passerine studies have also found that middle-ranked, rather than last-hatched, nestlings are often more susceptible to ectoparasites, given that ectoparasites face a trade-off between host resistance and nutritional quality, which might lead them to preferentially target nestlings of intermediate condition and immunocompetence [67].

In our experiment, all brood members were treated on the same day, rather than at a fixed age for each nestling, to maintain the natural brood size hierarchy under normal conditions and to minimise disturbance at the nest. If we had treated all brood members at the same age, then A and B nestlings would have been effectively treated two to four days earlier than C nestlings, giving them a further growth advantage over the one that occurs naturally. By treating on the same day, however, C nestlings received the treatment two to four days younger than A and B nestlings. One possibility is that C nestlings could benefit from being treated at an earlier stage of development than their counterparts, for example before heavy parasite burdens have developed. However, this would seem unlikely given that ivermectin acts by affecting the neurotransmitters of parasites that are present in the host and does not necessarily prevent reinfection; instead we might predict that A and B nestlings have more to gain from the treatment if burdens vary with nestling age, as they have had a longer period exposed to parasitic infective stages in the food. C nestlings were also on average ca. 60% smaller in mass than A and B nestlings when treated, but were administered the same absolute concentration of ivermectin. Thus C nestlings effectively received higher doses, which might have contributed to the observed growth rate differences. However, we could examine this possibility statistically by including mass-at-treatment as a covariate in the analyses. The effects of mass-at-treatment and nestling rank (i.e. A, B or C) were confounded to some degree, given that size hierarchies were defined based on initial masses, but mass variation was nonetheless present among nestlings of the same rank. The results showed that mass-at-treatment did not have a significant effect on nestling growth rates, controlling for the variables identified as having significant effects. Furthermore, the model including nestling rank and its interaction with treatment had an AIC that was lower by 12.06 units than a model including mass-at-treatment and its interaction with treatment, indicating significantly more relative support [68]. The data therefore suggest that growth rate differences among treated nestlings were indeed driven by chick rank effects.

In conclusion, the observation that anti-parasite treatment reversed growth rate patterns among family members in shags suggest that parasites play an important role in modulating sibling competition and family dynamics, which could have important consequences for offspring survival and reproductive success. Further work is required, however, to clarify the mechanisms by which growth rates of individual brood members are differentially affected by parasites.
